# Expansion cohorts in phase 1 oncology trials: a systematic review of their design, implementation and outcomes

**DOI:** 10.1038/s41416-025-03334-5

**Published:** 2026-01-10

**Authors:** Julio Herrero Colomina, Xinjie Hu, Habana Dinizulu, Ruiyang Yan, Ereny Poles, Rhona Dawson, Christina Yap, Louise Carter

**Affiliations:** 1 Experimental Cancer Medicine Team, The Christie NHS Foundation Trust, Manchester, UK; 2 Clinical Trials and Statistics Unit, The Institute of Cancer Research, London, UK; 3 Division of Cancer Sciences, University of Manchester, Manchester, UK

**Keywords:** Drug development, Randomized controlled trials

## Abstract

The urgent need for drug development for cancer patients and the complexity of novel therapies have led to the increasing importance of expansion cohorts (EC) within phase 1 trial design. We conducted a systematic review of oncology phase 1 trials with EC published from 2019 to 2023. The objective was to assess the characteristics, purpose and outcomes of EC. A mixed-effects meta-regression model was conducted to analyse response rates. 479 published phase 1 trials with EC were included (median number of EC patients per trial: 27). The EC objective was stated in 55.7% of studies (76.8% safety, 16.5% dosing, 25.8% pharmacokinetics, 22.1% pharmacodynamics, 77.5% preliminary efficacy). 117 trials (24.4%) included a statistical justification plan. The mean Overall Response Rate (ORR) was 20.2% in solid tumours and 46.8% in haematological malignancies. Among drug classes, Antibody-drug conjugates showed the highest ORR (32.1%). Higher ORR was significantly associated with combination therapies, haematological trials, trials with statistical justification for EC sample size and trials not containing Immunotherapy. EC have evolved to become large dynamic studies assessing both preliminary efficacy and safety. This study highlights the importance of clearly stated EC objectives and sample size justification to enhance the rigour and interpretability of early-phase evidence.

## Introduction

Phase 1 clinical trials mark the first time a new drug or combination is tested in humans. In oncology, these trials classically involve patients with advanced cancer who have exhausted other treatment options [1]. Traditionally, the primary objectives of these early phase trials are to assess safety and tolerability of a novel drug and to determine its optimal dose and schedule for use in further development [2]. They normally consist of a dose escalation part (often referred as Phase 1a), which is used to identify the optimal dose and a dose expansion part (often referred as Phase 1b), that focuses on further evaluating the safety, pharmacokinetics (PK), pharmacodynamics (PD) and preliminary efficacy at the chosen dose.

Originally, dose expansion cohorts were first incorporated into early phase trials to confirm the Recommended Phase 2 Dose (RP2D) in a larger patient cohort after a maximum Tolerated Dose (MTD) was reached during the dose escalation [1]. It reduced the uncertainty around the appropriate dose to investigate in subsequent phase 2 clinical trials [3]. These trials were not designed to evaluate efficacy but aimed to benefit patients therapeutically [4]. This approach fitted the old paradigm of cytotoxic trials, when there were few drugs for testing and toxicities were normally detected in the first weeks of treatment [3].

Between 1990 and 2014, an average of 9.1–9.4 years were required to receive US regulatory approval for oncology drug development [5]. However, in 2014 Pembrolizumab was approved by the FDA for melanoma patients just 3.4 years from the beginning of the KEYNOTE-001 trial [6]. This first-in-human, single-arm phase 1 clinical trial, which underwent nine protocol amendments based on interim analyses, enroled 1235 patients and ultimately led to the accelerated approvals of Pembrolizumab for melanoma and non-small cell lung cancer. This landmark trial paved the way for a subsequent evolution of phase 1 trial design. Modern studies aim to consolidate early phase trial stages through single repeatedly amended protocols to produce a more seamless trial design. They facilitate testing for different dose levels in specific indications analysing safety and preliminary efficacy in parallel resulting in recruitment of a larger patient population overall to the trial. This strategy has proven to be successful in numerous targeted and immune therapy trials [7] with some drugs receiving conditional approvals based on large expansion cohort results, significantly reducing drug development time [8].

Manji et al. first published a systematic review of expansion cohort use in single-agent phase 1 cancer trials published between 2006 and 2011 [1] noting 25% of published phase 1 trials included at least one dose expansion cohort [1]. Among the 149 phase 1 trials reviewed which included an expansion cohort, a median number of 17 patients were enroled into the dose expansion part of the studies. Objectives were clearly stated in 74% of these trials, with safety being the most common one (80%), followed by efficacy (45%), PK (28%) and PD (23%).

Norris et al. published in 2016 another systematic review of all phase 1 trials published in a single journal between 2004 and 2014. 93.3% of analysed trials were single agent studies and 78.6% of expansion cohorts tested a non-cytotoxic drug. They found that only 4% of studies provided a sample size justification for adding an expansion cohort. Also, the inclusion of an expansion cohort did not impact RP2D, subsequent phase 2 development or FDA approval.

Traditional small expansion cohorts in phase 1 trials substantially increased the probability of selecting the true MTD but they were not statistically powered to evaluate efficacy [9]. Nowadays, modern large expansion cohorts implemented to evaluate preliminary efficacy as well as safety need a sample size justification to justify their design and objectives, particularly when enrolment approached traditional phase 2 cohort sizes [3]. Expansion cohorts must have a clear purpose and a rigorous statistical analysis plan. This is supported by the recently published SPIRIT (Standard Protocol Items: Recommendations for Interventional Trials) Dose-finding Extension (DEFINE) and CONSORT (CONsolidated Standards Of Reporting Trials)-DEFINE guidelines to enhance transparency, comprehensiveness and consistency in early phase trials protocols and publications, respectively [10–12]. Every protocol amendment and extension should be preplanned and be powered to meet the study objectives [5]. In addition, protocols should consider inclusion of stopping rules to avoid patients from being treated with ineffective or unsafe doses or schedules [3]. We aimed to perform an updated systematic review 10 years after KEYNOTE-001 changed the paradigm of phase 1 trials to determine the objectives, characteristics and outcomes of dose expansion studies.

## Methods

### Data sources and searches

We conducted a systematic review of MEDLINE and EMBASE, focusing on oncology phase 1 trials with expansion cohorts involving adult participants published between the 1st of January 2019 and the 31st of December 2023 (Supplementary Material Fig. S1). Expansion cohorts were defined as additional patient groups treated with the same investigational treatment as the dose escalation, to further explore a selected dose.

### Study selection

Inclusion and exclusion criteria were defined before the online search. Manuscripts were selected for final analysis if they were prospective phase 1 clinical trials with expansion cohorts, recruiting adult participants and published between 2019 and 2023. Articles were excluded if: they included normal healthy volunteers, were not for the treatment of cancer, not investigating antineoplastic agents, were phase 2 studies (also excluding phase 1/2 trials to avoid heterogeneity), food effect studies, drug-drug interaction studies, ADME studies, radiotherapy trials, radioembolization, nuclear medicine trials, stem-cell or bone-marrow transplantation or supportive care trials without antineoplastic agent. In addition, studies which only reported long term survival, non-English publications, conference abstracts and duplicate manuscripts were also excluded from the systematic review.

Two reviewers (JH and HD) assessed the titles and abstracts of publications identified using the search strategy and any publication considered potentially relevant was selected. Subsequently, the same two reviewers evaluated the complete publications for eligibility. In cases where it was unclear whether a study met inclusion or exclusion criteria, the final determination was made by a third reviewer (LC).

### Objectives

The objective of this systematic review was to assess the characteristics, purpose and outcomes of phase 1 expansion cohorts. The purpose for conducting an expansion cohort in the phase 1 trial was identified from the ‘Methods’ section of manuscripts. All the studies which did not clearly justify the aim of expanding the trial protocol in the methods were classified as ‘purpose not clearly indicated’. The statistical justification plan for opening an expansion cohort such as statistically powered sample size calculation was also gathered from the ‘Methods’ section.

Data on safety, PK, PD and efficacy were gathered by noting whether they were reported alongside dose escalation data or separately. Information regarding the expansion cohort adding new toxicity data was included. Assessment of preliminary efficacy was done by analysing the Overall Response Rate (ORR) (complete and partial responses) and disease control rate (DCR) (complete, partial and stable disease responses) reported in the articles as per RECIST 1.1 criteria [13]. Trials adding new preliminary efficacy data were defined as those studies in which at least one additional partial or complete response was seen in the expansion cohort.

### Data extraction

Data extraction from the published manuscripts for included trials was performed by five of the authors (JH, HD, RY, RD, EP) using a standardised spreadsheet for data collection. Consensus was achieved in cases of uncertainty by involvement of another reviewer (LC). Characteristics of phase 1 clinical trials with expansion cohorts extracted from the manuscripts can be found in the Supplementary Methods. Further details on the data extraction are provided in the Supplementary Material.

### Data synthesis and statistical analysis

Descriptive analyses of the characteristics and purposes of expansion cohorts were performed using SPSS 26.0. The primary outcomes were ORR and DCR. To focus on studies where preliminary efficacy outcomes were predominately informed by the expansion cohorts, we included (i) studies which reported expansion data separately and (ii) studies which combined escalation and expansion data but where the expansion cohort comprised >75% of the total sample size.

Meta-analysis was conducted in R version 4.3.1 using the metafor package [14]. A mixed-effects meta-regression model was applied, beginning with univariable analysis, followed by a multivariable model. All independent variables with a *p* < 0.05 in univariable analyses were included in the multivariable model. Study-level covariates are detailed in the Supplementary Materials. Results are reported as regression coefficients (β), 95% CIs and *p* values, with statistical significance defined as *p* < 0.05. A comprehensive description of the Data Synthesis and Statistical Analysis can be found in the Supplementary Material.

## Results

**Fig. 1:**
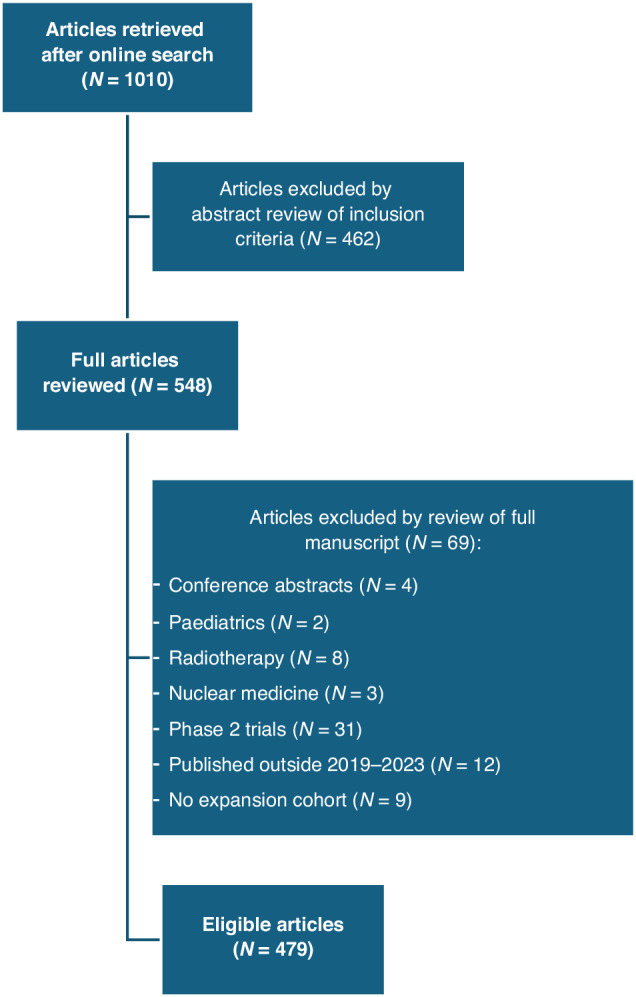
Flow diagram of study selection.

In total, 1010 articles were retrieved from the online search. Of these, 479 met all the inclusion and exclusion criteria and were considered eligible after being reviewed by the authors (Fig. 1).

### Characteristics of phase 1 studies with expansion cohorts

**Fig. 2:**
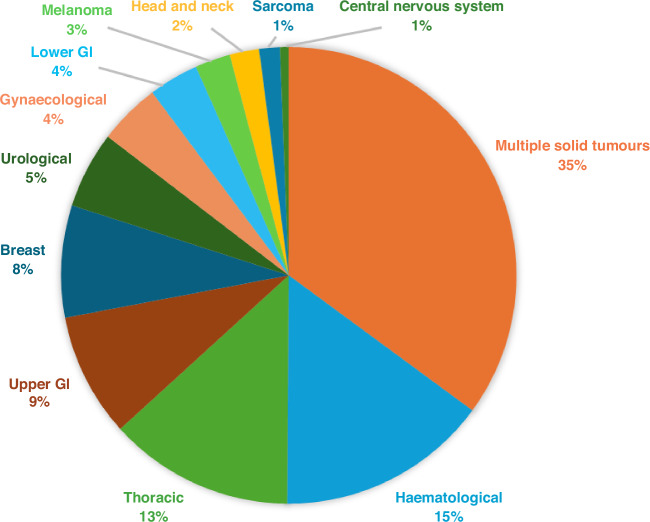
Tumour subtypes’ distribution in phase 1 expansion trials (479 studies). GI gastrointestinal.

The phase 1 trials included in this analysis, detailed in Table 1, were published between 2019 and 2023 and encompassed 18,995 patients across the expansion cohorts, with a median number of expansion cohort patients per trial of 27 (interquartile range: 38). The vast majority of expansion trials were multicentric (88.9%) and funded by the industry (83.9%). Targeted therapies constituted 43.2% of all investigational drugs tested (rising to 68.3% when combined with other agents); Immunotherapy, either in monotherapy or in combination, accounted for 23.6% of all treatments; and antibody-drug conjugates (ADCs), either alone or in combination, accounted for 9% of the total. The complete table with different classes of agents can be found in Supplementary Table S1. With regards to the drug administration, intravenous therapies were the most common route of delivery comprising 63% of the total (Supplementary Table S2). Many different tumour subtypes were recruited to these trials, with haematological tumours accounting for 15% and thoracic tumours being the most common type among solid tumours at 13.2% (Fig. 2). There were 168 trials (35%) which included multiple advanced solid tumours in their eligibility criteria, 112 of which were for all-comers.

**Table 1:** Characteristics of phase 1 clinical trials with expansion cohorts

Characteristics	Trials with expansion cohorts (*N* = 479)	
	No.	%
Patients enrolled in escalation		
Median	22	
Interquartile Range	24	
Patients enrolled in expansion		
Median	27	
Interquartile Range	38	
Sponsor		
Academic	77	16.1
Industry	402	83.9
Centre		
Uni-centre	53	11.1
Multi-centre	426	88.9
Continent		
North America	310	64.7
Asia	188	39.2
Europe	177	37
Oceania	38	7.9
Combination		
Monotherapy	248	51.8
Combination therapy	231	48.2
Specialty		
Solid tumours	407	85
Haematological tumours	72	15
Class of agent		
Included chemotherapy	94	19.6
Included ADC	43	17.3
Included Immunotherapy	110	23
Included Targeted therapy	327	68.3
Dose finding strategy		
Rule based	383	80
Model based	52	10.9
Not stated	44	9.2
Reported phase designation		
Phase 1	329	68.7
Phase 1b	111	23.2
Phase I/1b	16	3.3
Phase 1a/1b	23	4.8

Expansion cohorts were published together with the dose escalation cohorts in 426 studies (88.9%). In terms of study design, 192 trials (40.1%) had different inclusion criteria for the dose expansion cohorts compared to the dose escalation, whereas 287 trials (59.9%) reported similar population criteria for both escalation and expansion.

184 trials (38.4%) required patients to have the presence of specific biomarkers as part of the trial eligibility. Alterations in EGFR were the most common targeted molecular alterations required for trial eligibility in expansion cohorts (41 trials), followed by HER2 (16 trials) and the RAS/RAF/MEK/ERK (16 trials) and PTEN/PI3K/AKT/mTOR (14 trials) pathways. A summary of the biomarkers required for eligibility in expansion cohorts of the trials is included in Supplementary Table S3.

### The purpose of expansion cohorts in phase 1 clinical trials

The objective of including the expansion cohorts was clearly stated in 55.7% of studies (76.8% safety, 16.5% dose refinement, 25.8% PK, 22.1% PD and 77.5% efficacy) whilst for the remaining 44.3% of studies it was not clearly defined. With regards to sample size justification of the expansion cohort, only 117 trials (24.4%) included a statistical plan supporting the addition of further patients after dose escalation to meet trial objectives.

### The outcomes of expansion cohorts in phase 1 clinical trials

Safety and toxicity were reported in 471 studies (98.3%). 276 trials (57.6%) presented dose escalation and dose expansion data all together, whereas 195 (40.7%) published the dose expansion data separately. Of this last group, authors from 125 expansion trials (26.5%) reported new toxicity data, defined as emergent adverse event terms that had not been identified in the dose escalation or increased severity of previously identified toxicities. PK information was published in 299 articles (62.4%), with 91 of them (19%) reporting dose expansion data independently. In addition, 251 studies (52.4%) incorporated PD data, of which 209 (43.7%) reported the expansion cohort information separately.

Preliminary efficacy was reported in 470 studies (98.1%). 234 trials (48.9%) published dose escalation and dose expansion data all together, whereas 236 (49.3%) reported the dose expansion data separately. Of this last group, 167 studies (35.5%) added new efficacy data, defined as one or more partial or complete responses in the expansion cohort.

**Fig. 3:**
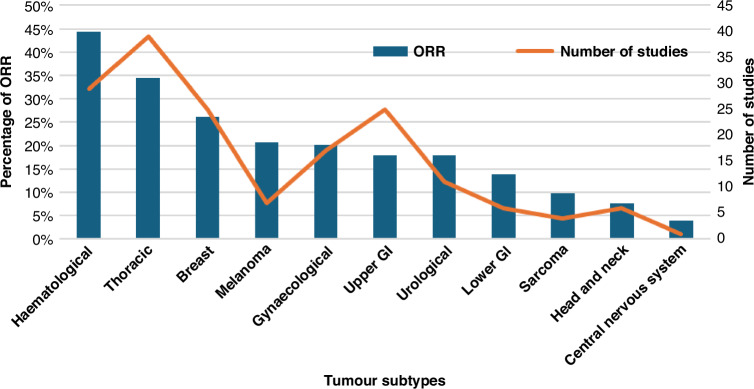
Overall Response Rate (ORR) (blue bars) and number of phase 1 expansion trials (orange line) per tumour subtype in trials focused on a specific tumour type which reported the expansion cohort ORR separately (266 trials out of 479 trials). GI gastrointestinal, ORR Overall Response Rate.

The mean ORR was 20.2% for studies recruiting solid tumour patients and 46.8% for haematological patients, whereas the DCR was 56.4% for solid tumour trials and 62.8% for haematological trials. Amongst solid tumours, thoracic and breast trials showed the highest ORR with 34.4% and 26.2% respectively. Response rate among tumour subtypes is summarised in Fig. 3. Detailed information about ORR and DCR by tumour subtypes is described in Supplementary Table S4.

Amongst the different types of therapies, trials testing ADCs showed the highest mean ORR and DCR with 32.1% and 70.6%, respectively. Trials which included immunotherapy showed a 16.9% ORR and 50.3% DCR, whereas trials containing targeted therapies showed 21.5% ORR and 55.1% DCR, respectively. In 25 trials (5.2%), the RP2D was modified after the expansion. Additionally, 20 trials (4.2%) terminated development of the drug following the expansion cohort. Furthermore, 15 studies (3.1%) were prematurely closed for reasons other than trial completion. According to the ClinicalTrials.gov database, 283 of the reviewed studies (59.1%) advanced to later-stage trials by the time of this analysis.

Finally, we performed a mixed-effect meta-regression model to investigate factors associated with higher ORR and DCR. The results of the univariate and multivariate analyses were consistent. For the latter, combination trials (*p* = 0.0324), trials with a statistical justification for the expansion cohort (*p* = 0.0038), haematology trials (*p* < 0.0001) and trials not containing Immunotherapy (*p* = 0.0103) were significantly associated with higher ORR (Supplementary Tables S5 and S6). In addition, trials which included a statistical justification for the expansion cohort (*p* = 0.0099) and those without Immunotherapy (*p* = 0.0024) were significantly associated with higher DCR (Supplementary Tables S7 and S8).

## Discussion

This is the first systematic review in the past decade to analyse the characteristics, purpose and outcomes of dose expansion cohorts in phase 1 clinical trials. The characteristics of phase 1 expansion trials have evolved markedly over the last 15 years to adapt to drug development needs with increasing emphasis on early assessment of preliminary efficacy. The median number of patients enroled has increased substantially, from 17 patients in Manji’s review to 27 in this study [1]. Although this does not reach the typical sample size of phase 2 trials, usually between 70 and 140 participants [15], this marked increase requires justification in each study, particularly due to the increased uncertainties around dose and toxicities patients in phase 1 trials are exposed to.

The proportion of novel therapies investigated has increased, though this is driven by trials of immunotherapies rather than targeted agents (5% and 72%, respectively, in the Norris review versus 21% and 75% in our analysis) [2]. Also, we have witnessed the emergence of ADC as a new way of delivering cytotoxic compounds to cancer patients. There has only been a small increase in the use of model-based dose finding strategies, from 6–8% to 10% of trials in our review [16, 17]. These novel strategies have the potential to facilitate more efficient dose escalation trials gathering patients’ data in real time, but their increased complexities and costs associated with statistician time may be limiting their uptake [7].

There are some features in expansion trials, however, that have remained stable over the years. The proportion of haematology trials compared to those recruiting solid tumours continues to represent approximately one in six studies. Some tumour subtypes, such as central nervous system, sarcomas or head and neck cancers, continue to represent the lowest percentage of all solid tumours recruited to phase 1 trials [1]. It is interesting to note that they are also precisely the tumour types with lowest response rates in our review. The poorer outcomes seen with patients with rarer cancers highlights the need for increased investment in drug development for orphan diseases and therefore it is disappointing to see the continued low numbers of studies for these tumour types [18].

Overall, only 38.4% of the reviewed trials incorporated a biomarker as an eligibility criterion. In the era of precision oncology, this figure remains disappointingly low. Greater efforts are needed to advance biomarker development, enabling more refined patient selection and ultimately potentially enhancing the efficacy of novel therapies [19].

To our knowledge, this is the first systematic review of expansion-phase 1 trials that specifically analyses response rates. We identified four key factors associated with increased ORR in phase 1 trials with expansion cohorts, namely tumour type recruited, combination versus monotherapy trials, class of agents tested and statistical justification for the expansion cohort. Haematology trials consistently demonstrated higher response rates compared to solid tumour trials, likely due to well-established biological and pharmacological differences [20]. In addition, combination therapy trials were shown to yield higher response rates than monotherapy trials consistent with previous studies [21].

Trials that did not involve immunotherapies were also associated with higher ORR. The interpretation of this finding is less straightforward due to the heterogeneity of immunotherapy trials included. However, it may suggest that current strategies to target the immune system—beyond checkpoint inhibition—have yet to demonstrate consistent efficacy in early-phase settings, potentially due to the lack of reliable biomarkers. This contrasts with the high response rate of ADCs in the present review, which represent one of the more successful strategies in drug development over the past decade.

Of note trials that incorporated a statistical justification for the expansion cohort were associated with both higher ORR and DCR. In contrast, the univariate analyses showed that number of patients included in the expansion cohort did not correlate with increase ORR or DCR. It is interesting to see that studies with clearly reported statistical plans are also those able to objectively demonstrate increased response rate, underscoring the importance of including a robust statistical rationale when designing expansion cohorts.

The drive towards assessing efficacy early in phase 1 trials as opposed to a focus on safety alone is reflected in the stated objectives of expansion cohorts: 76.8% of trials in our review identified safety as an objective for the expansion cohort, whereas 77.5% reported preliminary efficacy as the main reason to recruit more patients to the trial. Manji et al. reported similar percentages for safety, but only 45% of studies in their review aimed to look at efficacy in the expansion cohort. It is also, concerning to note that the proportion of transparently reported objectives has decreased from 74% to 55.7% compared to Manji’s review [1]. Given the growing complexity of early-phase clinical trials, careful protocol design and transparent, prospective reporting of study objectives are critical to ensuring the validity and credibility of result interpretation [10, 11, 22].

Encouragingly, however, given the increased importance placed on efficacy assessment in dose expansion studies there has been an increase in the number of phase 1 studies providing a statistical plan to support sample sizes. While only 4% of expansion cohorts in the Norris’ review were supported by a statistical rationale [2], 24.4% of trials in the present review reported a statistical justification supporting the addition of new patients to meet trial objectives such as safety, PK/PD or preliminary efficacy. Although statistical justification has improved over the past decade, considerable progress is still needed to adequately support the growing numbers of patients enroled in these cohorts. Statistical justification is essential to ensure expansion cohorts are designed to avoid exposing patients to futile and potentially harmful therapies, and to enable robust assessment of the main objectives of early phase trials.

The changing focus for the objectives of dose expansion studies is reflected in the reported data. Manji et al. reported that 54% of trials contributed new safety data during the expansion phase, and 13% led to modifications of the RP2D [1]. In contrast, our study found that only 26.5% of trials reported novel safety findings, and just 5.2% resulted in dose adjustments following the expansion cohort. The reason for this change is likely multifactorial, but it may be explained by better-designed dose escalation trials, which help investigators identify limiting toxicities early on. From an efficacy standpoint, 33.5% of trials in Manji’s review reported tumour responses, with only 18.1% distinguishing between data from the dose-escalation and expansion phases [1]. In comparison, 98.1% of the trials included in our review reported preliminary efficacy outcomes, and 49.3% clearly differentiated between escalation and expansion efficacy results.

PK and PD data was highly heterogeneous across studies and often not clearly reported. As a result, data on discordance between escalation and expansion PK/PD were not systematically collected. Future early-phase studies should consistently report PK and PD data separately for the initial and expansion cohorts. This would allow assessment of any differences between cohorts and ensure transparency in interpreting how expansion cohort data align with initial dose-finding results, ultimately supporting more informed dose selection and trial design decisions.

The increased focus on assessing efficacy at earlier and earlier stages in drug development influences the objectives, characteristics and outcomes of dose expansion studies being delivered now compared to 15 years ago. Initially conceived as small protocol extensions aimed at confirming the safety profiles of novel drugs, they have transformed into large, dynamic and increasingly complex cohorts primarily focused on assessing efficacy. This evolution stems from several factors. Firstly, there has been a paradigm shift in phase 1 trials from cytotoxic drugs to modern targeted therapies and immunotherapies, characterised by distinct toxicity profiles and response rates and trends [21]. Secondly, given the urgent need to expedite drug development timelines for cancer patients there have been significant changes in the regulatory landscape with the advent of breakthrough therapy designation, fast track designation and accelerated approval pathways [23]. Finally, the emergence of new statistical models that guide the enrolment of patients in phase 1 trials has accelerated dose escalation and increased the recruitment numbers of expansion cohorts.

This review has a number of limitations. We excluded expansion cohorts from phase 1/2 and phase 2 trials to minimise heterogeneity given the lack of consensus in reporting the name of early-phase studies; however, this may have led to the omission of valuable insights from phase 1/2 studies with larger patient populations. Additionally, conference abstracts were excluded because they often do not have sufficient methodological or outcome details to permit reliable data extraction and appraisal. While this approach strengthened the quality and consistency of included evidence, it may have led to omission of some information from ongoing trials. Much like many early phase trials, not all studies with ECs are published, which may introduce publication bias and limit the generalisability of our findings [24]. The fact that non-English studies were excluded from the review may limit generalisability of findings to global trial landscape. Although the analysis is robust, its retrospective nature limits the direct applicability of the conclusions. Finally, the trials included in this review are highly heterogeneous. While the study offers meaningful insights into trends in expansion cohorts in phase 1 clinical trials, the numerical results should be interpreted with caution.

In conclusion, phase 1 expansion cohorts have evolved markedly over the past decade. Contemporary expansion cohorts often enrol substantially more patients and frequently include multiple study arms with the aim to evaluating not only safety, but also PK, PD and particularly preliminary efficacy. Our review shows that the vast majority of treatments under investigation are now novel targeted therapies and immunotherapies. Objectives of expansion cohorts are less explicitly stated than in the past, and there is a noticeable shift towards prioritising efficacy endpoints. Although statistical planning has improved, considerable progress is still needed to ensure robust justification for the increasing scale and complexity of these cohorts. In line with the new SPIRIT-DEFINE and CONSORT-DEFINE guidelines, investigators should clearly specify adaptive features, sample size rationale and the dose expansion strategy [10]. Our findings reinforce the necessity of a pre-specified statistical plan to maximise the scientific value of expansion cohorts, protect patient interests and strengthen the overall interpretability of early phase trial outcomes. Expansion cohorts in early phase oncology trials can inform key development decisions, including dose refinement and early termination of unpromising agents, while informing progression to later-phase studies. Careful planning, including clear objectives and pre-specified statistical analyses, appears to enhance their value. Standardised reporting and prospective evaluation will be important to quantify their contribution and optimise their role in clinical development.

## Supplementary information

Supplementary material

## Data Availability

The data underlying this article are available in Mendeley Data: Herrero, Julio (2025), ‘Characteristics and outcomes of expansion cohorts in phase 1 oncology trials: a systematic review’, V1, 10.17632/hrp3mwjmh8.1.

## Reporting guidelines

This study was written in accordance with the PRISMA guidelines (see Supplementary Material check list).
