# Supplementary material for: Expansion cohorts in phase 1 oncology trials: a systematic review of their design, implementation and outcomes
Source: Br J Cancer. 2026 Jan 10;134(8):1131–7. doi: 10.1038/s41416-025-03334-5 (PMC13036038; doi:10.1038/s41416-025-03334-5)
Supplement: Supplementary file 1 — Supplementary material [file 41416_2025_3334_MOESM1_ESM.pdf]

Supplementary Material

Supplementary Methods

Data extraction

Characteristics of phase 1 clinical trials with EC included year of publication, trial location (continent), number of centres involved (uni- or multi-centre), sponsor nature (academia vs industry), drug name, class of agent, type of administration, monotherapy vs combination trials, solid vs haematological tumours, number of patients enrolled and dose finding strategy (rule-based vs model-based). Whether the purpose of the phase 1 EC, (safety/toxicity, dose refinement, PK, PD, efficacy) was clearly described and whether there was a statistical justification for the sample size provided was also extracted.

The characteristics of the EC including tumour types and tumour families recruited, molecular alterations required for eligibility, number of patients enrolled, patient population (same target population as dose escalation or different) were also extracted. The outcomes of the EC including toxicity reporting (together with dose escalation data or separately), emergent toxicity data, PK and PD reporting (together with dose escalation data or separately), efficacy reporting (together with dose escalation or separately), emergent efficacy data, response rate (proportion of patients with complete or partial responses), disease control rate (proportion of patients with complete response, partial response or stable disease), Recommended Phase 2 Dose (RP2D) modified after EC, whether the EC led to termination of a drug's development, premature closing of EC for other reasons and subsequent development of the drug as per further trials identified with the investigational medicinal product(s) (IMP) in clinicaltrials.gov. were noted.

Data Synthesis and Statistical Analysis

A random-effects meta-analysis was first conducted using the restricted maximum likelihood method to estimate pooled risk ratios with 95% confidence intervals (CIs). Between-study heterogeneity was assessed using the I^2 statistic and Cochran's Q-test, with I^2 values above 50% considered indicative of substantial heterogeneity. When majority of efficacy outcomes are < 0.2 or > 0.8, the logistic transformed data - log risk ratios will be used in the meta-regression model; otherwise, raw data will be used.

To identify potential sources of heterogeneity and study-level covariates of treatment effect, a mixed-effects meta-regression model was fitted, initially in the univariable and then in the multivariable setting. All independent variables with a p value less than 0.05 in univariable analysis were included in the multivariable model. Study-level covariates included 1) whether or not the study has targeted molecular alteration as an inclusion criterion for the expansion cohort; 2) whether or not the study is a combination trial ; 3) whether or not the drug continued to be developed to a phase 2 or 3; 4) whether or not purpose of the expansion cohort was stated at Methods; 5) whether or not there was a statistical plan for justifying the number of patients enrolled at the expansion cohort; 6) whether or not the trial included solid tumours; 7) whether or not the trial contained immunotherapy; 8) whether or not the trial contained targeted therapies; 9) number of patients in the expansion cohort. Covariates were entered as fixed effects, while a random-effects term was retained to account for residual heterogeneity.

The summary effect size for each subgroup is computed using a random-effects model with between-study and within-study variance components. In this project, we attribute the differences in these observed within-group variance estimates solely to sampling error, hence we anticipate a common between-study variance component across different subgroups. We will pool between-study variance for subgroups in the model to calculate within-group estimates and

identify the potential source of heterogeneity indicated by study-level covariates with p value less than 0.05.

From S3-S6 we can see that the significant factors indicated in the univariable analysis are also significantly related with ORR/DCR in the multivariable analysis, hence the conclusion we have drawn from univariable setting aligns with what we obtain from multivariable setting, which indicates that the applied model is robust and well explained the relationship between the trial characteristics and ORR/DCR.

Figure S1. Search Strategy in MEDLINE and EMBASE

Database: Embase <1974 to 2024 January 18>, Ovid MEDLINE(R) ALL <1946 to January 18, 2024>

1 ("phase i" or "phase 1").ti,ab. (196774)
2 clinical trial, phase i/ or phase 1 clinical trial/ (105603)
3 1 or 2 (221565)
4 expansion.ti,ab. (493874)
5 3 and 4 (12554)
6 exp neoplasms/ or exp neoplasm/ or (cancer* or neoplasm* or tumo?r* or malign*).mp. [mp=ti, ab, hw, tn, ot, dm, mf, dv, kf, fx, dq, bt, nm, ox, px, rx, ui, sy, ux, mx] (12622349)
7 5 and 6 (11174)
8 limit 7 to yr="2019 - 2023" (6274)
9 limit 8 to (article or journal article) [Limit not valid in Embase,Ovid MEDLINE(R); records were retained] (1827)
10 remove duplicates from 9 (1010)

Table S1. Class of agent investigated in phase 1 trials with expansion cohorts. Abbreviations: IO (Immunotherapy), TT (Targeted therapy), ADC (Antibody Drug Conjugate)

Class of Agent	Number of Trials	Percentage of Trials (%)
Chemotherapy	23	4.8
Immunotherapy	57	11.9
Targeted therapy	207	43.2
ADC and other conjugates	37	7.7
Hormonal therapy	6	1.3
Cell therapy	9	1.9
Vaccines	6	1.2
Chemotherapy + IO	4	.8

Chemotherapy + TT	61	12.7
IO + TT	45	9.4
Hormonal + TT	10	2.1
Hormonal + IO	1	.2
Others	2	.4
ADC + Chemotherapy	2	.4
ADC + IO	2	.4
ADC + TT	2	.4
Chemotherapy + IO + TT	2	.4
Vaccine + IO	2	.4
Vaccine + Chemotherapy	1	.2
Total	479	100.0

Table S2. Route of Drug Administration in Phase I Trials with Expansion Cohorts

Route of Drug Administration	Number of trials	Percentage of trials
iv	204	42.6
oral	166	34.7
iv and oral	92	19.2
oral and im	3	.6
sc	3	.6
iv and im	4	.8
sc and iv	2	.4
sc and oral	2	.4
intradermal	1	.2
intraperitoneal	1	.2
intratumoral	1	.2
Total	479	100.0

Table S3. Number of Trials with Molecular Alterations Required for Eligibility

Molecular alterations	For the whole study (escalation and expansion)	For the expansion cohort exclusively
EGFR	25	16
Her-2	14	2
RAS/RAF/MEK/ERK	7	9
PTEN/PI3K/AKT/mTOR	10	4
MET	5	7
FGF	2	6

ALK	7	0
Cyclin family	6	1
IDH	6	0
ROS	4	0
KIT	3	0
BRCA	1	2

Table S4. Response Rate and Disease Control Rate among Tumour Families.

Tumour Types	Response Rate	Number of trials (Total: 266)*	Disease Control Rate	Number of trials (Total: 240)*
Breast	26.2%	25	57%	24
Central Nervous System	4%	1	NA	0
Gynaecological	20.1%	17	60.9%	13
Haematological	44.3%	29	60.1%	22
Head And Neck	7.76%	6	45.3%	6
Lower GI	13.8%	6	51.7%	6
Melanoma	20.6%	7	50.5%	7
Multiple	14.7%	96	49.1%	89
Sarcoma	9.87%	4	56.5%	4
Thoracic	34.4%	39	71.3%	37
Upper GI	17.9%	25	60.4%	23
Urological	17.9%	11	67.8%	9

* Trials with non-missing response rate and disease control rate in the published manuscript.

Table S5: Univariable mixed-effects meta regression results for objective response rate (ORR). Logit-transformed ORRs are modelled by study-level covariates. Coefficients represent the estimated effect of each covariate on the log-odds of response. Predicted effects, corresponding to ORRs, are back-transformed probabilities with 95% confidence intervals from the logit scale. Covariates with $p<0.05$ are considered statistically significant and highlighted in red.

Univariable Analysis - ORR	Meta regression		
	Logic transformation		
	Predicted effect (CI 95%)	Coefficients	P-value
Targeted molecular alteration as an inclusion criterion for the expansion cohort (W)	0.184 (0.160, 0.210)	-0.0897	0.7067
No (W=0)	0.186 (0.160, 0.214)		
Yes (W=1)	0.172 (0.120, 0.243)		
Combination trials (Q)	0.187 (0.130, 0.260)	0.4356	0.0092
No (Q=0)	0.156 (0.129, 0.187)		
Yes (Q=1)	0.222 (0.183, 0.267)		
Continued development of the drug to a phase 2 or 3 (AW)	0.181 (0.145, 0.223)	0.2696	0.1186
No (AW=0)	0.160 (0.127, 0.199)		
Yes (AW=1)	0.200 (0.168, 0.235)		
Purpose/outcome of the expansion cohort stated at Methods. If not clearly stated, add 1 to "Not clear purpose" (AI)	0.184 (0.160, 0.210)	0.0157	0.9279
Clear purpose (AI=0)	0.183 (0.154, 0.216)		
Not clear purpose (AI=1)	0.185 (0.148, 0.229)		
Was there a statistical plan for justifying the number of patients enrolled at the EC? (AJ)	0.193 (0.135, 0.269)	0.4394	0.0132
No (AJ=0)	0.163 (0.137, 0.192)		
Yes (AJ=1)	0.232 (0.185, 0.286)		
Includes solid tumours, or haematological malignancies only (R)	0.289 (0.084, 0.644)	-1.5246	<.0001
Only haematological tumours (R=0)	0.469 (0.363, 0.578)		
Includes solid tumours (R=1)	0.161 (0.141, 0.184)		
Trials containing Immunotherapy (M)	0.175 (0.127, 0.237)	-0.3883	0.0328
No (M=0)	0.202 (0.173, 0.235)		
Yes (M=1)	0.147 (0.113, 0.188)		
Trials containing targeted therapies (N)	0.184 (0.160, 0.210)	-0.0506	0.7764
No (N=0)	0.189 (0.149, 0.236)		

Univariable Analysis - ORR	Meta regression		
	Logic transformation		
	Predicted effect (CI 95%)	Coefficients	P-value
Yes (N=1)	0.181 (0.153, 0.213)		
Number of patients in the expansion cohort (Y)	0.182 (0.148, 0.223)	0.0001	0.9375

Table S6: Multivariable mixed-effects meta regression results for objective response rate (ORR) with variables which were significant in the univariate analysis. Logit-transformed ORRs are modelled by study-level covariates. Coefficients represent the estimated effect of each covariate on the log-odds of response. Predicted effects, corresponding to ORRs, are back-transformed probabilities with 95% confidence intervals from the logit scale. Covariates with p<0.05 are considered statistically significant and highlighted in red.

Multivariable Analysis -ORR	Meta regression	
	Logic transformation	
	Coefficients	P-value
Targeted molecular alteration as an inclusion criterion for the expansion cohort (W)	Not included	
Combination trials (Q)	0.3332	0.0324
Continued development of the drug to a phase 2 or 3 (AW)	Not included	
Purpose/outcome of the expansion cohort stated at Methods. (AI)	Not included	
Was there a statistical plan for justifying the number of patients enrolled at the EC? (AJ)	0.4686	0.0038
Includes solid tumours, or haematological malignancies only (R)	-1.3654	<.0001
Trials containing Immunotherapy (M)	-0.4378	0.0103
Trials containing targeted therapies (N)	Not included	
Number of patients in the expansion cohort (Y)	Not included	

Table S7: Univariable mixed-effects meta regression results for disease control rate (DCR). Logit-transformed DCRs are modelled by study-level covariates. Coefficients represent the estimated effect of each covariate on the log-odds of response. Predicted effects, corresponding to DCRs, are back-transformed probabilities with 95% confidence intervals from the logit scale. Covariates with p<0.05 are considered statistically significant and highlighted in red.

Univariable Analysis - DCR	Meta regression		
	No transformation		
	Predicted effect (CI 95%)	Coefficients	P-value
Targeted molecular alteration as an inclusion criterion for the expansion cohort (W)	0.568 (0.537, 0.598)	0.0239	0.5717
No (W=0)	0.564 (0.531, 0.597)		
Yes (W=1)	0.588 (0.512, 0.664)		
Combination trials (Q)	0.570 (0.512, 0.629)	0.0598	0.0547
No (Q=0)	0.541 (0.501, 0.582)		
Yes (Q=1)	0.601 (0.556, 0.647)		
Continued development of the drug to a phase 2 or 3 (AW)	0.568 (0.537, 0.598)	0.0258	0.4219
No (AW=0)	0.552 (0.502, 0.602)		
Yes (AW=1)	0.578 (0.539, 0.616)		
Purpose/outcome of the expansion cohort stated at Methods. (AI)	0.571 (0.524, 0.619)	0.0487	0.1268
Clear purpose (AI=0)	0.549 (0.511, 0.588)		
Not clear purpose (AI=1)	0.598 (0.549, 0.647)		
Was there a statistical plan for justifying the number of patients enrolled at the EC? (AJ)	0.578 (0.512, 0.645)	0.0682	0.0426
No (AJ=0)	0.547 (0.511, 0.584)		
Yes (AJ=1)	0.615 (0.560, 0.670)		
Includes solid tumours, or haematological malignancies only (R)	0.592 (0.507, 0.678)	-0.0909	0.0838
Only haematological tumours (R=0)	0.650 (0.552, 0.748)		
Includes solid tumours (R=1)	0.559 (0.527, 0.591)		
Trials containing Immunotherapy (M)	0.551 (0.463, 0.638)	-0.0891	0.0099
No (M=0)	0.592 (0.557, 0.627)		
Yes (M=1)	0.503 (0.445, 0.561)		
Trials containing targeted therapies (N)	0.568 (0.537, 0.598)	0.0232	0.4908
No (N=0)	0.552 (0.497, 0.607)		
Yes (N=1)	0.575 (0.538, 0.612)		
Number of patients in the expansion cohort (Y)	0.567 (0.522, 0.612)	0.0000	0.9634

Table S8: Multivariable mixed-effects meta regression results for disease control rate (DCR) with variables which were significant in the univariate analysis. Logit-transformed DCRs are modelled by study-level covariates. Coefficients represent the estimated effect of each covariate on the log-odds of response. Predicted effects, corresponding to DCRs, are back-

transformed probabilities with 95% confidence intervals from the logit scale. Covariates with p<0.05 are considered statistically significant and highlighted in red.

Multivariable Analysis -DCR	Meta regression	
	Logic transformation	
	Coefficients	P-value
Targeted molecular alteration as an inclusion criterion for the expansion cohort (W)	Not included	
Combination trials (Q)	Not included	
Continued development of the drug to a phase 2 or 3 (AW)	Not included	
Purpose/outcome of the expansion cohort stated at Methods. (AI)	Not included	
Was there a statistical plan for justifying the number of patients enrolled at the EC? (AJ)	0.0866	0.0099
Includes solid tumours, or haematological malignancies only (R)	Not included	
Trials containing Immunotherapy (M)	-0.1054	0.0024
Trials containing targeted therapies (N)	Not included	
Number of patients in the expansion cohort (Y)	Not included	

Table 9. PRISMA (Preferred Reporting Items for Systematic Reviews and Meta-Analyses) checklist

Section and Topic	Item #	Checklist item	Location where item is reported
**TITLE**			
Title	1	Identify the report as a systematic review.	1
**ABSTRACT**			
Abstract	2	See the PRISMA 2020 for Abstracts checklist.	2
**INTRODUCTION**			
Rationale	3	Describe the rationale for the review in the context of existing knowledge.	4-5
Objectives	4	Provide an explicit statement of the objective(s) or question(s) the review addresses.	6, 8
**METHODS**			
Eligibility criteria	5	Specify the inclusion and exclusion criteria for the review and how studies were grouped for the syntheses.	7

Section and Topic	Item #	Checklist item	Location where item is reported
Information sources	6	Specify all databases, registers, websites, organisations, reference lists and other sources searched or consulted to identify studies. Specify the date when each source was last searched or consulted.	7
Search strategy	7	Present the full search strategies for all databases, registers and websites, including any filters and limits used.	7 and supplementary materials
Selection process	8	Specify the methods used to decide whether a study met the inclusion criteria of the review, including how many reviewers screened each record and each report retrieved, whether they worked independently, and if applicable, details of automation tools used in the process.	7
Data collection process	9	Specify the methods used to collect data from reports, including how many reviewers collected data from each report, whether they worked independently, any processes for obtaining or confirming data from study investigators, and if applicable, details of automation tools used in the process.	8
Data items	10a	List and define all outcomes for which data were sought. Specify whether all results that were compatible with each outcome domain in each study were sought (e.g. for all measures, time points, analyses), and if not, the methods used to decide which results to collect.	Supplementary materials
	10b	List and define all other variables for which data were sought (e.g. participant and intervention characteristics, funding sources). Describe any assumptions made about any missing or unclear information.	Supplementary materials
Study risk of bias assessment	11	Specify the methods used to assess risk of bias in the included studies, including details of the tool(s) used, how many reviewers assessed each study and whether they worked independently, and if applicable, details of automation tools used in the process.	8

Section and Topic	Item #	Checklist item	Location where item is reported
Effect measures	12	Specify for each outcome the effect measure(s) (e.g. risk ratio, mean difference) used in the synthesis or presentation of results.	8
Synthesis methods	13a	Describe the processes used to decide which studies were eligible for each synthesis (e.g. tabulating the study intervention characteristics and comparing against the planned groups for each synthesis (item #5)).	7
	13b	Describe any methods required to prepare the data for presentation or synthesis, such as handling of missing summary statistics, or data conversions.	8 and supplementary materials
	13c	Describe any methods used to tabulate or visually display results of individual studies and syntheses.	8
	13d	Describe any methods used to synthesize results and provide a rationale for the choice(s). If meta-analysis was performed, describe the model(s), method(s) to identify the presence and extent of statistical heterogeneity, and software package(s) used.	9 and supplementary materials
	13e	Describe any methods used to explore possible causes of heterogeneity among study results (e.g. subgroup analysis, meta-regression).	9 and supplementary materials
	13f	Describe any sensitivity analyses conducted to assess robustness of the synthesized results.	9 and supplementary materials
Reporting bias assessment	14	Describe any methods used to assess risk of bias due to missing results in a synthesis (arising from reporting biases).	9 and supplementary materials
Certainty assessment	15	Describe any methods used to assess certainty (or confidence) in the body of evidence for an outcome.	9 and supplementary materials
**RESULTS**			
Study selection	16a	Describe the results of the search and selection process, from the number of records identified in the search to the number of studies included in the review, ideally using a flow diagram.	10 and supplementary materials

Section and Topic	Item #	Checklist item	Location where item is reported
	16b	Cite studies that might appear to meet the inclusion criteria, but which were excluded, and explain why they were excluded.	Supplementary materials
Study characteristics	17	Cite each included study and present its characteristics.	10
Risk of bias in studies	18	Present assessments of risk of bias for each included study.	16
Results of individual studies	19	For all outcomes, present, for each study: (a) summary statistics for each group (where appropriate) and (b) an effect estimate and its precision (e.g. confidence/credible interval), ideally using structured tables or plots.	10-12 and supplementary materials
Results of syntheses	20a	For each synthesis, briefly summarise the characteristics and risk of bias among contributing studies.	10-12 and supplementary materials
	20b	Present results of all statistical syntheses conducted. If meta-analysis was done, present for each the summary estimate and its precision (e.g. confidence/credible interval) and measures of statistical heterogeneity. If comparing groups, describe the direction of the effect.	12 and supplementary materials
	20c	Present results of all investigations of possible causes of heterogeneity among study results.	Supplementary materials
	20d	Present results of all sensitivity analyses conducted to assess the robustness of the synthesized results.	Supplementary materials
Reporting biases	21	Present assessments of risk of bias due to missing results (arising from reporting biases) for each synthesis assessed.	16
Certainty of evidence	22	Present assessments of certainty (or confidence) in the body of evidence for each outcome assessed.	12 and supplementary materials
**DISCUSSION**			
Discussion	23a	Provide a general interpretation of the results in the context of other evidence.	13-16
	23b	Discuss any limitations of the evidence included in the review.	16

Section and Topic	Item #	Checklist item	Location where item is reported
	23c	Discuss any limitations of the review processes used.	16
	23d	Discuss implications of the results for practice, policy, and future research.	16-17
**OTHER INFORMATION**			
Registration and protocol	24a	Provide registration information for the review, including register name and registration number, or state that the review was not registered.	Not registered
	24b	Indicate where the review protocol can be accessed, or state that a protocol was not prepared.	Protocol is not available
	24c	Describe and explain any amendments to information provided at registration or in the protocol.	N/A
Support	25	Describe sources of financial or non-financial support for the review, and the role of the funders or sponsors in the review.	18
Competing interests	26	Declare any competing interests of review authors.	18
Availability of data, code and other materials	27	Report which of the following are publicly available and where they can be found: template data collection forms; data extracted from included studies; data used for all analyses; analytic code; any other materials used in the review.	18
